# Supplementary material for: Theta-phase dependent neuronal coding during sequence learning in human single neurons
Source: Nat Commun. 2021 Aug 10;12:4839. doi: 10.1038/s41467-021-25150-0 (PMC8355141; doi:10.1038/s41467-021-25150-0)
Supplement: Supplementary file 1 — Supplementary Information [file 41467_2021_25150_MOESM1_ESM.pdf]

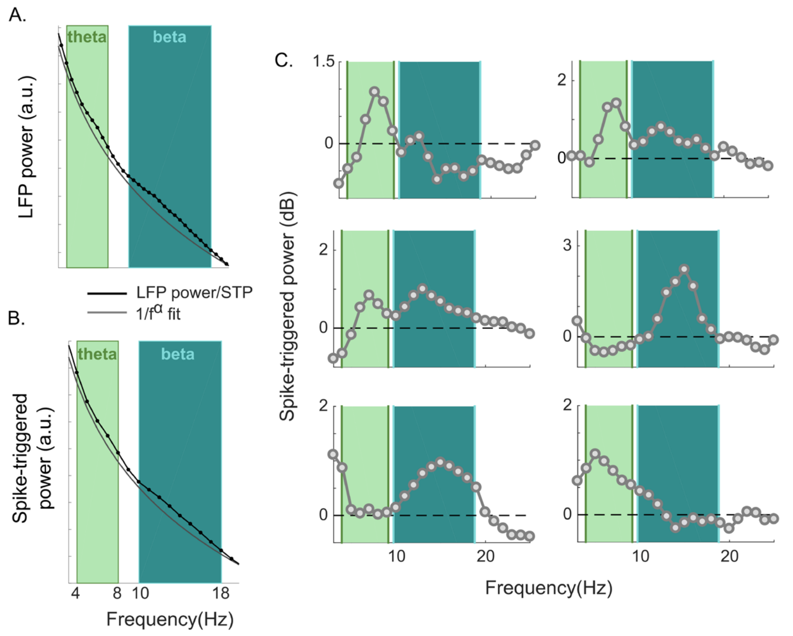

Supplementary Figure 1. A) Pre-whitened LFP power. B) Pre-whitened spike-triggered power (STP). C) Individual cell examples of STP (after subtraction of the $1/f^{\alpha}$ fit).

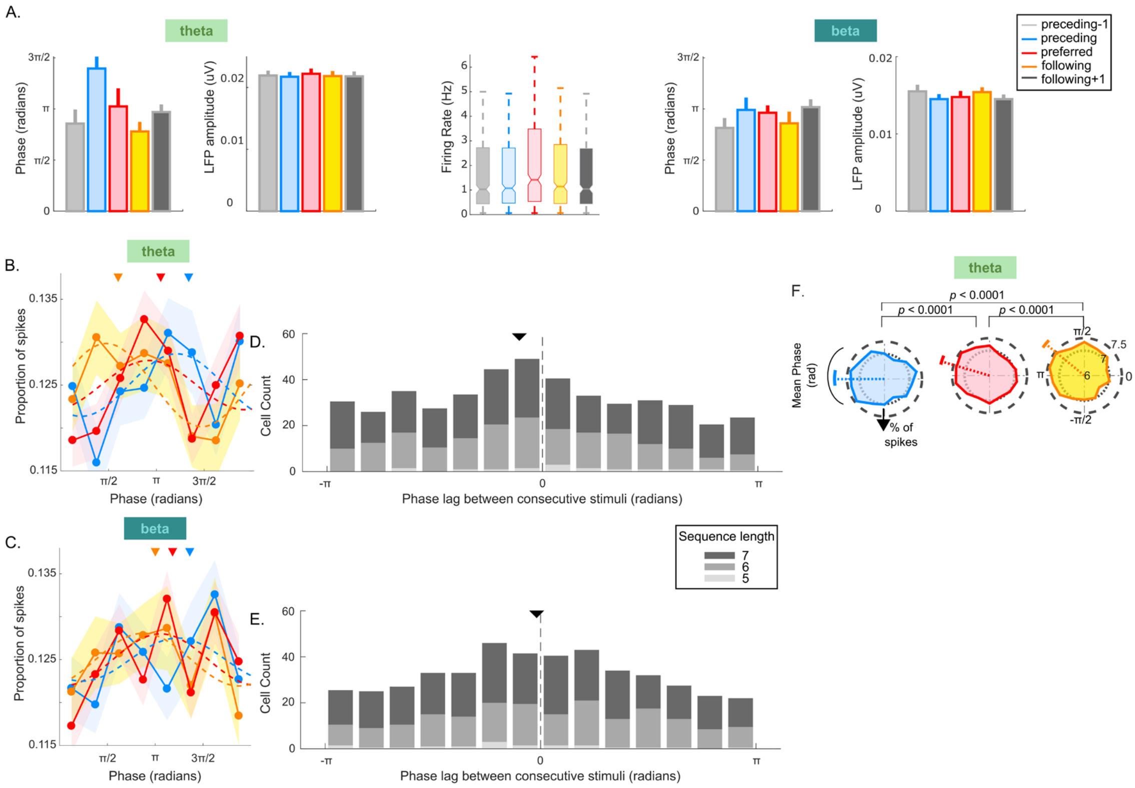

Supplementary Figure 2. A) Differences in firing rates and LFP amplitudes do not account for phase precession. Phase, LFP amplitude (square root of LFP power), and firing rates are shown for the preceding-1 (light gray), preceding (blue), preferred (red), following (yellow), and following+1 (dark gray) stimuli, in the theta (left column) and beta (right column) bands. The largest phase difference was observed for the preceding and following stimuli in the theta band even though the firing rates and LFP amplitudes were not different (phase difference between the preceding and following stimuli, Watson-William Test, $F(1,902) = 74.8$; $p<0.0001$, significant after Bonferonni correction for 10 pairwise comparisons of five stimulus types; firing rate difference, $p=0.8$, paired t-test, two-sided; LFP amplitude difference, $p=0.8$, paired t-test, two-sided). The firing rate differences were significant for all pairs of stimuli (all $p<0.005$ uncorrected) except for preceding-1 vs. following+1, and preceding vs. following. The LFP amplitudes were not significantly different for all pairs of stimuli (all $p>0.05$, paired t-test) in both frequency bands. In the theta band, the phase differences were significantly different for all pairs of stimuli (Watson-William Test, all $p<0.005$ uncorrected), except for preceding-1 vs. following+1 ($p=0.02$), preceding-1 vs. following, and preferred vs. following+1 $p>0.05$). The vertical bars for the phase plots correspond to the mean ± one standard deviation across cells ($N = 452$ cells). In the box and whisker plots, the central mark indicates the median firing rate across cells, the notch reflects the 95% confidence interval for the median, and the box reaches from the first to the third quartile (interquartile range). In the LFP plots the vertical bars correspond to the mean ± standard error of the mean across cells. B, C) Unsmoothed distributions of spike phases relative to the theta and beta band LFPs for the preceding (blue), preferred (red), and following (yellow) stimuli. The format of these panels is the same as Figure 3 A, C). The dashed lines correspond to a sinusoidal fit. D, E) Distribution of phase lags between consecutive stimuli in the (D) theta (circular median test against 0 phase lag, $p=0.01$) and (E) beta bands ($p=0.6$). The distribution is gray scale-coded by the length of the sequence (there were 17 sessions with seven stimuli, 13 sessions with six stimuli, and one session with five stimuli). The black arrow indicates the mean phase lag value (-19.3 degrees in the theta band, -3.3 degrees in the beta band). This value in the theta band is smaller than the value obtained at the population level (Figure 3). This is because cells that have consistent phase-locking without any phase precession would show near-zero phase lags in this plot, but may be pointing in any direction in the population rose plots. F) Circular histogram when pooling all spikes from the population ($N= 449$, i.e., 2 cells with firing rate greater than 5 standard deviations above the mean firing rate of the population were removed since outlier cells with exceptionally high firing rates could dominate the outcome). The dashed colored lines indicate the mean phase, and the colored angular lines correspond to one standard deviation. The p-values above the rose plots are the results of a two-sample Watson-William test for equal means. Results of the Watson-William test: ($F(1,99475) = 198.8$; $p<0.0001$ for preferred vs. preceding; $F(1,99605) = 404.9$; $p<0.0001$ for preferred vs. following; and $F(1,90939) = 990.7$; $p<0.0001$ for preceding vs. following).

Following pages: Supplementary Figures 3-8 A,B. Examples of individual cells showing phase-precession. The format is the same for all cell examples.

a) 3-seconds of raw and filtered (4-8Hz) LFP traces in a single example trial. The tick marks correspond to simultaneously recorded spikes.

b) Raster plots and instantaneous firing rates for the preceding (blue), preferred (pink), and following (yellow) stimuli. The lighter areas correspond to the 0.5s inter-stimulus interval (ISI). The darker areas correspond to the 1.5s image presentation window. Image onset occurred at time=0s. For each cell, the preferred stimulus was the one that elicited the largest number of spikes across all repetitions of the sequence, and the consistency of stimulus preference was verified with a cross-validation analysis. The consistency score from the cross-validation analysis is reported in the yellow panel (see the methods section for a description of the cross-validation analysis and the consistency score). For all cells shown here, the consistency score was significantly higher than chance (0.5, one-sided Binomial test, $p<0.001$). The black curves and grey shaded areas correspond to the mean and standard error of the mean of firing activity across trials. For determining stimulus preferences all repetitions of each stimulus were included in the analysis, excluding the probe trials. Since the probe events occurred randomly, the number of non-probe trials for each stimulus can be different, resulting in different numbers of trials in the raster plots.

c) Phase distribution of spikes in the 4-8Hz theta band for this cell. The significance was determined by a Rayleigh test.

d) Scatter plot of theta phase versus time. For visualization purposes the phase values are copied vertically three times. The red line corresponds to the mean phase as a function of time as explained in panel f).

e) Heat map corresponding to the data shown in panel d). The scalebar represents the expected firing rate (Hz) at each point. For visualization purposes the data are copied vertically three times. The red line corresponds to the mean phase as a function of time as explained in panel f).

f) Change in phase for this cell as a function of time. For display purposes only, the phase value that is plotted in the black curve is the circular mean phase computed in sliding windows of 2.5s (shifted by steps of 0.112s, i.e. 50 steps in each figure). The larger black points and error bars correspond to the mean and standard deviation of the phase in (non-overlapping) bins for the preceding and following stimuli, plotted at the center of the 2.5s window in which the mean phase was computed. The phase lag value (in units of radians/stim) reported at the top of the plot is calculated from these non-overlapping bins as follows: $Phase\ Lag = (mean_{prec} - mean_{foll})/2$. In panels d, e, and f), time = 0s is the start of the preferred stimulus, time = -2s is the onset of the preceding stimulus, and time=2s is the onset of the following stimulus. Stimulus onsets are indicated by the thicker vertical lines in panels e) and f), and the ISI onsets are indicated by the thinner vertical lines.

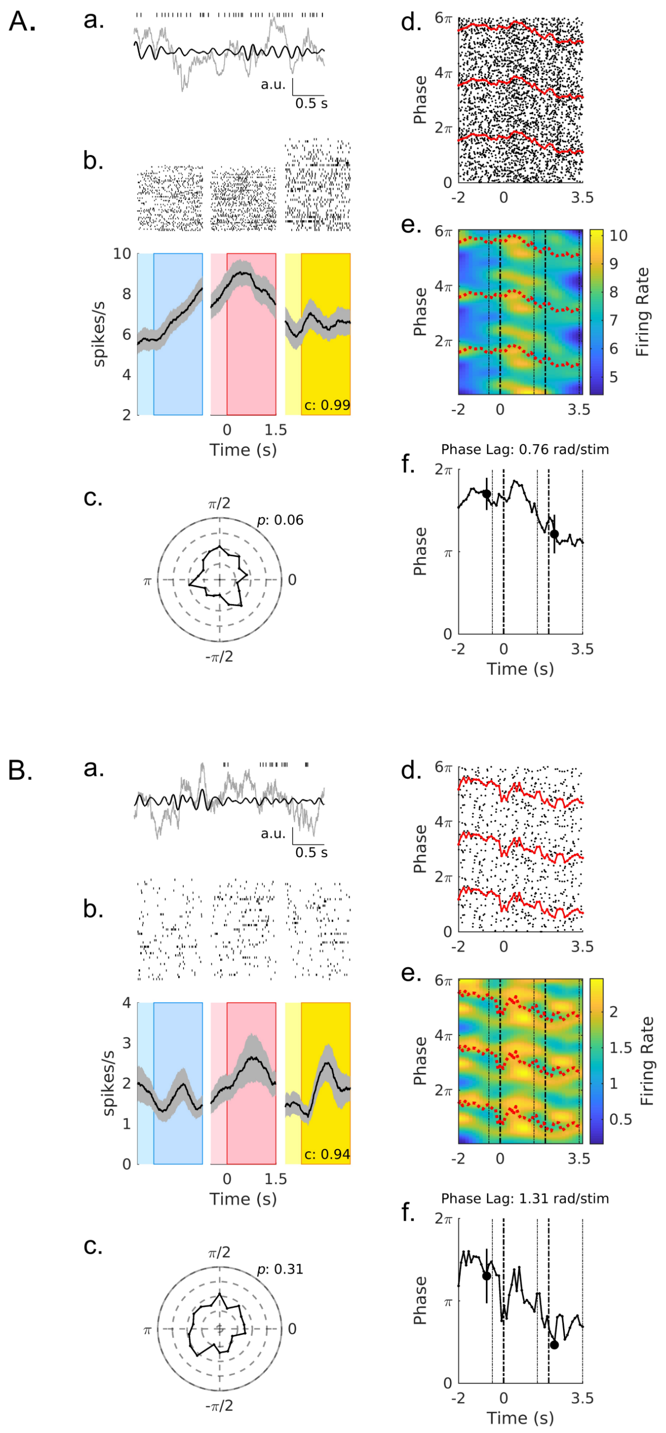

Supplementary Figure 3.

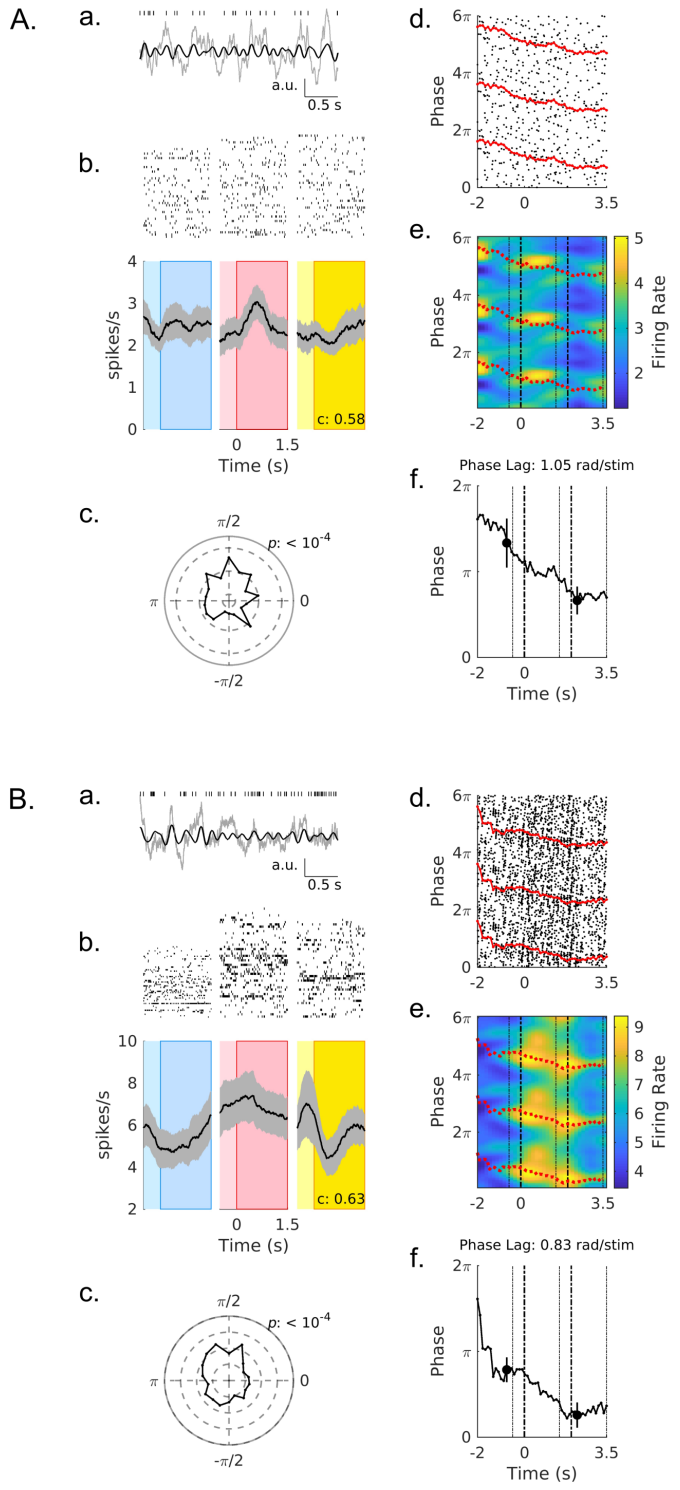

Supplementary Figure 4.

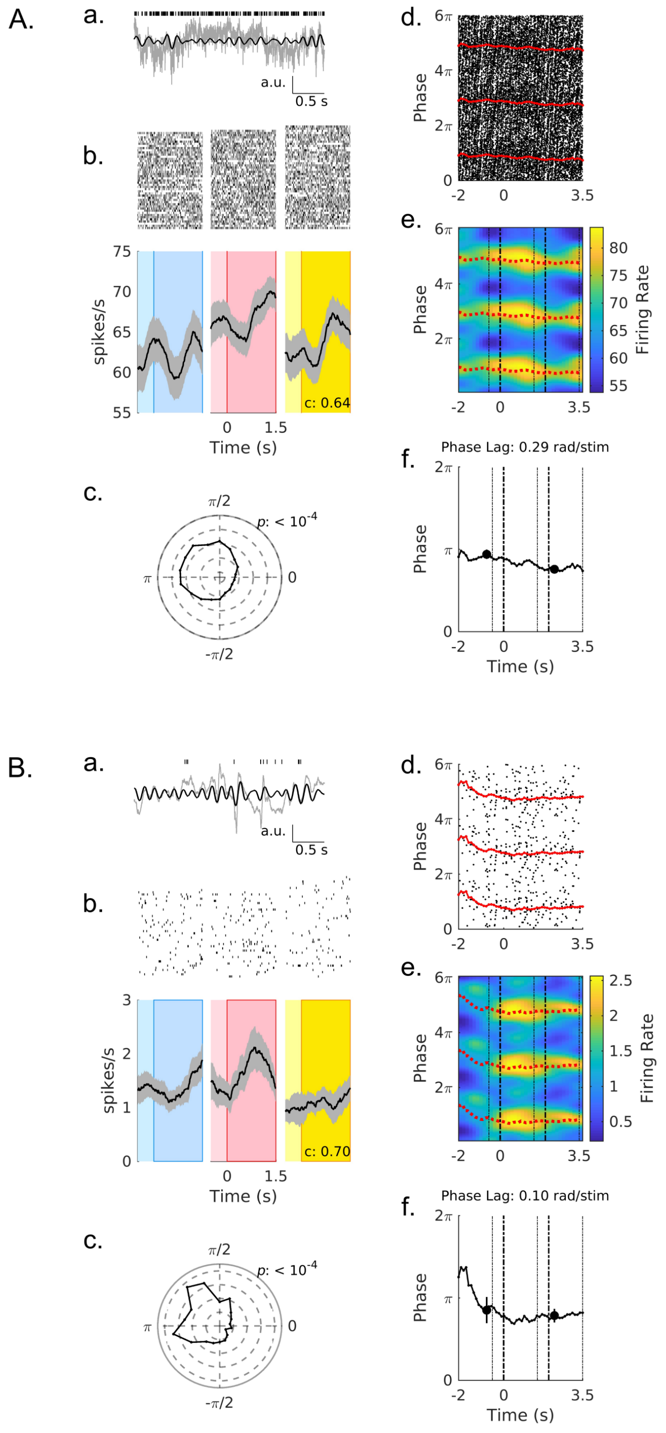

Supplementary Figure 5.

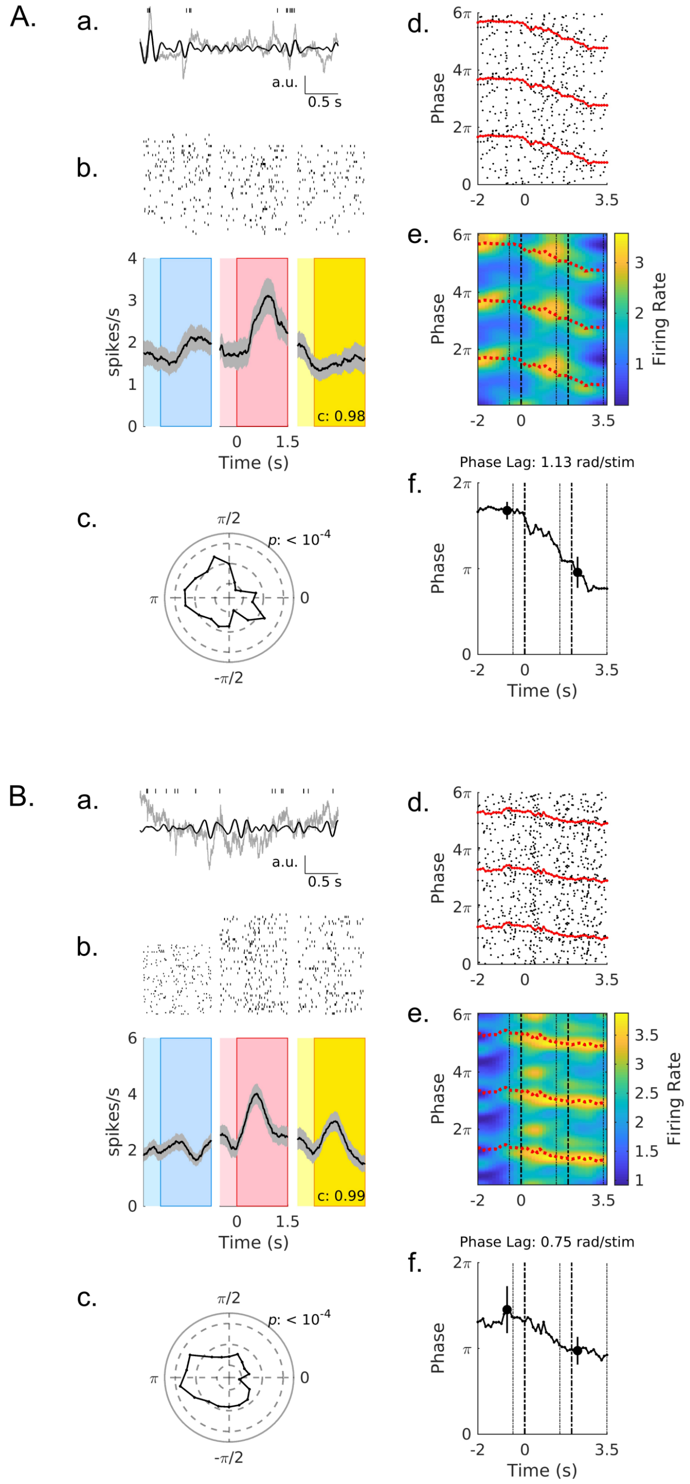

Supplementary Figure 6.

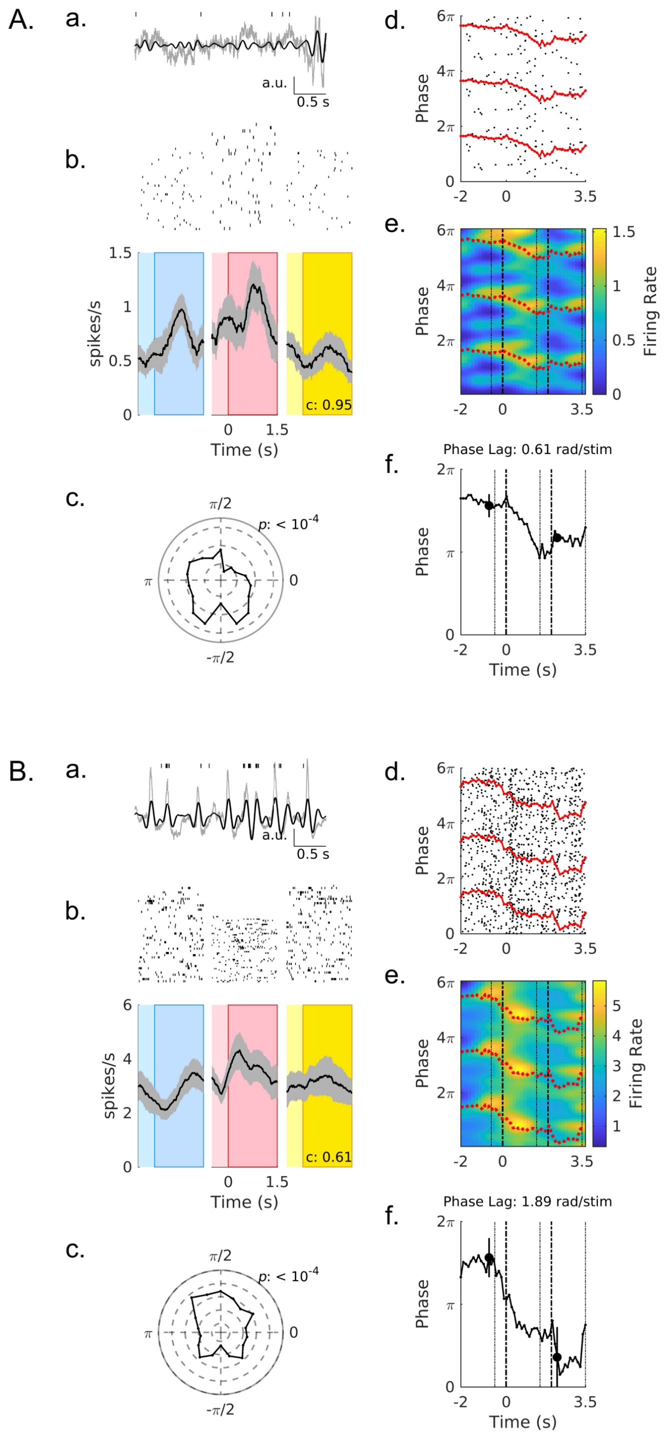

Supplementary Figure 7.

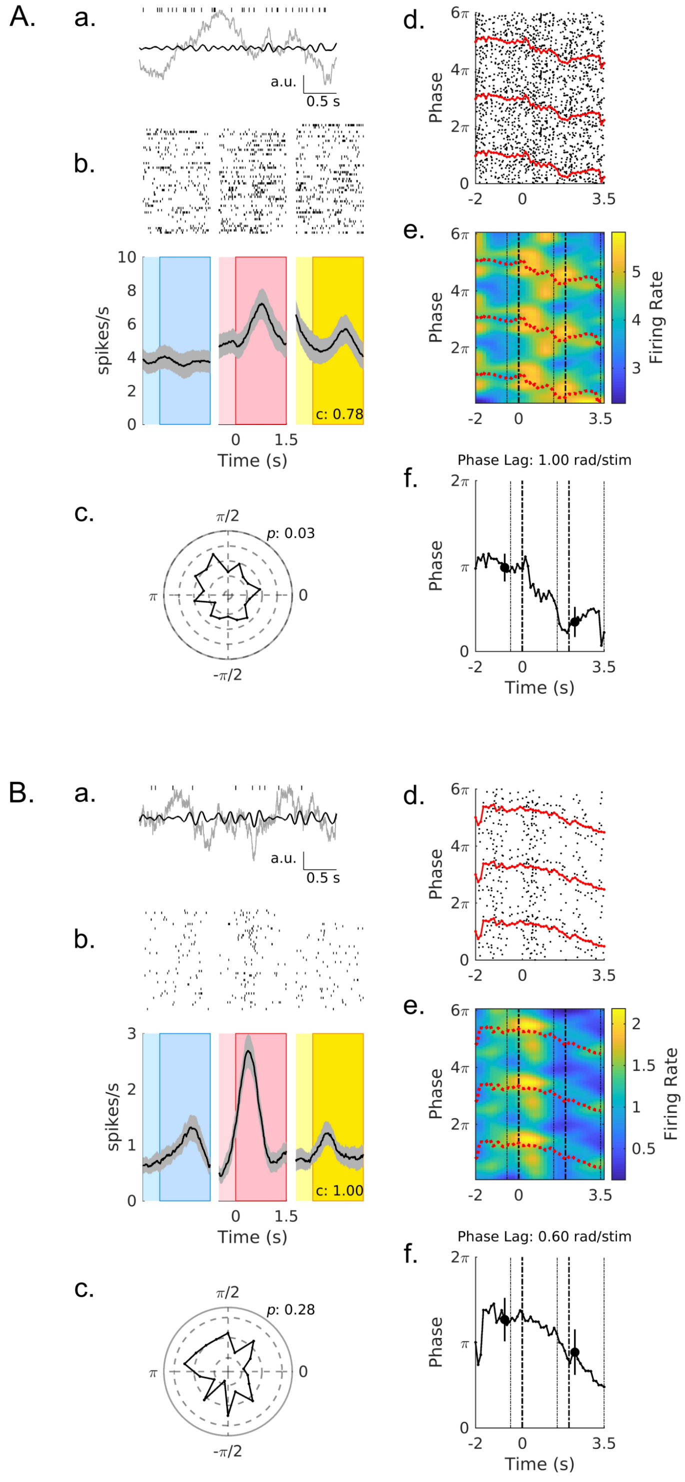

Supplementary Figure 8.

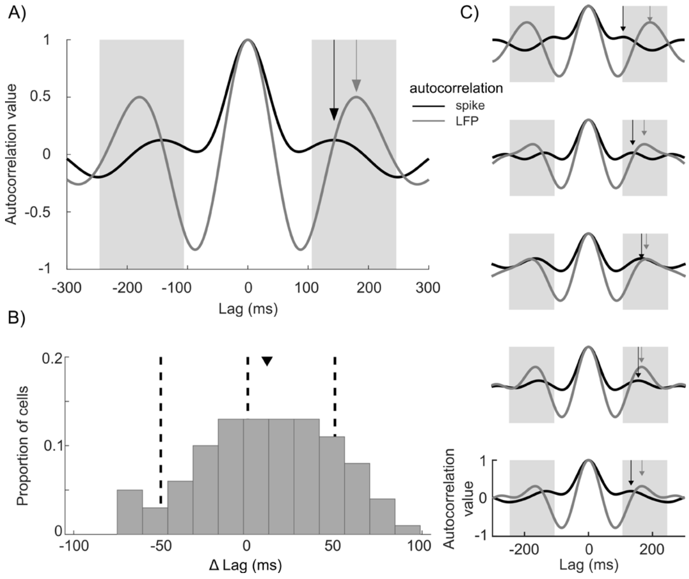

Supplementary Figure 9. A. Autocorrelations of spikes and LFPs. A) Representative example of the autocorrelation of a neuron and the LFP from the same recording site. The autocorrelation function was computed for the spikes (smoothed with a 25ms standard deviation Gaussian window) and for the LFP filtered in the theta-band. Peaks in the autocorrelations were detected in a time window of ± 70ms around the peak of the average LFP auto-correlogram across all cells, which was 178ms (this window has been indicated in grey). Note that for this cell, the first side peak for the spikes (black arrow) occurs at a shorter lag than the first side peak of the LFP autocorrelation function. In other words, the frequency of the oscillation is slightly higher for the spikes, as is expected during phase precession. B) Distribution of the difference between the peak lag of the LFP and the peak lag of the spikes across cells. The mean of the distribution (indicated by the black arrowhead) is shifted significantly to the right of 0 (mean ± sem = 11.02 ± 3.8 ms; 95% confidence interval of the mean = [3.4 18.7], $t(1,99) = 2.9$, $p < 0.01$, t-test against 0). To increase the reliability of the measure, only cells (N=100) that showed a clear theta-band peak in the autocorrelograms for both spikes and LFPs were included (i.e., the difference between the detected peak and the minimum of the autocorrelation in the highlighted time window was greater than a threshold of 0.15). C) Individual cell examples of the autocorrelation of spiking activity and the LFP.

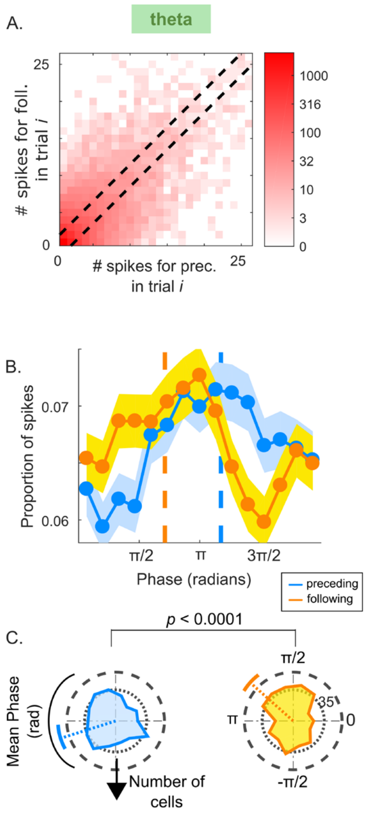

Supplementary Figure 10. Control for the possible influence of firing rate differences on phase precession measures. A. The spike count elicited by the preceding and following stimuli was matched at the level of individual trials. Specifically, we only included trials in which the firing rate difference was <=1 spike. The density plot shows the number of spikes fired in response to the preceding and following stimuli on each trial *i*. The analysis presented in this figure only uses trials that lie between the two dashed diagonal lines, i.e., trials on which the difference between the two stimuli was <=1 spike (N=422 cells remained). The mean and standard deviation of trials per cell was 14.5 ± 6.9 trials (see Methods).

B. Distribution of spike phases relative to the theta band LFP for the preceding (blue), and following (yellow) stimuli when using trials with matched firing rates (between the two dashed diagonal lines in panel A; N = 422). The dashed colored lines indicate the mean phase, the shaded areas correspond to the *SEM* across cells.

C. Firing phase across all cells *(N* = 422*),* with colors as in panel (B). The format of panels B and C is identical to that of Figure 3 (now only showing preceding and following stimuli, for which the spike numbers have been equated). The p-value above the rose plots is the result of a two-sample Watson-William test for equal means. Source data are provided as a source data file.

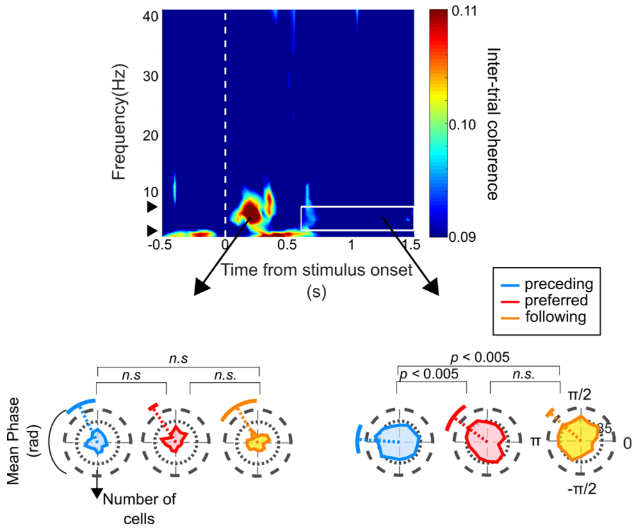

Supplementary Figure 11**.** Influence of phase-reset and inter-trial coherence (ITC) on phase precession. (*top*) The ITC quantifies the degree of phase locking of the LFP to stimulus onset. It was computed at each time and frequency point with respect to the onset of the preceding, preferred, and following stimuli. The black arrowheads on the y-axis correspond to the 4-8Hz frequency band. The phase reset caused by stimulus onset is visible as an increase in ITC, with a peak around 7Hz, at 250ms.

(*bottom left)* The phase calculated in a 60ms window around peak ITC is not significantly different between the preceding, preferred and following stimuli ($p > 0.1$; $N = 213$ cells).

(bottom right) Phase precession in a time window after 600 ms post-stimulus onset, during which ITC has subsided ($N = 446$ cells). In this window, phase differences were significant for the preceding vs. preferred and preceding vs. following stimuli ($F(1,890) = 8.7$; $p<0.005$ for preceding vs. preferred, uncorrected; $F(1,890) = 10.8$; $p<0.005$ for preceding vs. following, uncorrected; and $F(1,890) = 0.7$; $p>0.05$ for preferred vs. following; Watson-William test). Source data are provided as a source data file.

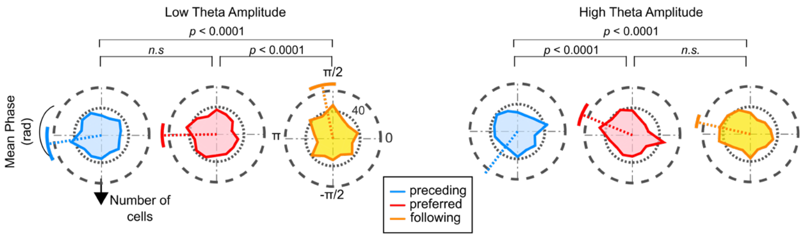

Supplementary Figure 12. Spike-phase coupling during periods of A) low and B) high theta amplitude ($N = 452$ cells). We determined the median of the distribution of theta amplitudes of each recording channel. Spikes for each cell were then assigned to periods of low or high theta on the basis of the instantaneous theta power at the moment of the spike (median split). Within each period we then recomputed the phase of firing for the stimuli in the sequence. The rose plots show the distributions of spike phases for the preceding (blue), preferred (red) and following (yellow) stimuli. Phase differences were evident during epochs of low and high theta for the extreme stimuli (preceding vs. following). Low theta: $F(1,902) = 65.1$; $p<0.0001$ for preceding vs. following; $F(1,902) = 59.2$; $p<0.0001$ for preferred vs. following; and $F(1,902) = 0.4$; $p>0.05$ for preceding vs. preferred; Watson-William test, uncorrected. High-theta: $F(1,902) = 16.3$; $p<0.0001$ for preceding vs. following; $F(1,902) = 1.3$; $p>0.05$ for preferred vs. following; and $F(1,902) = 27.1$; $p<0.0001$ for preceding vs. preferred. Source data are provided as a source data file.

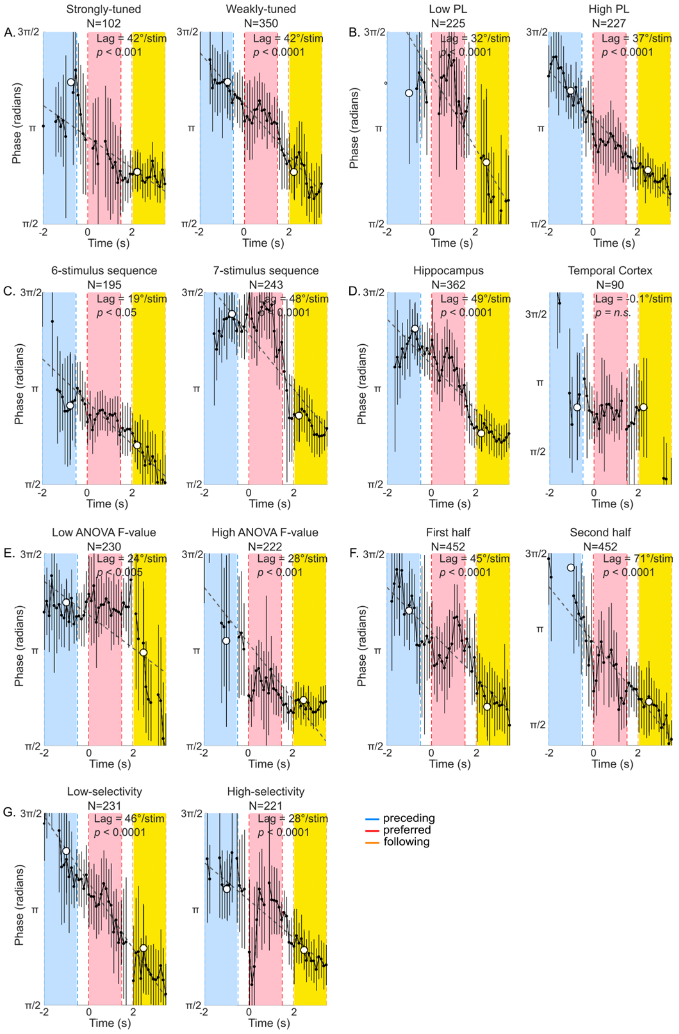

Supplementary Figure 13. Strength of phase precession examined in different conditions. In all panels, for display purposes only, the small black dots and lines correspond to the circular mean and standard deviation of phase values across cells, plotted as a function of time in sliding windows (Methods). The circular mean was only plotted when the requirements for confidence limits were met. The mean phase was computed in sliding time windows; as described in the Methods section in panels where the data were reduced by a median split (B, E-G), the window size was 2s, and 2.5s for the remaining panels. The large white points and error bars correspond to the mean and standard deviation of the phase in (non-overlapping) bins for the preceding and following stimuli, plotted at the center of the time window in which the mean phase was computed. The phase lag value (in units of degrees/stim) reported at the top of each panel is calculated from these non-overlapping bins as follows: $Phase\ Lag = (mean_{prec} - mean_{foll})/2$. Significance of the phase lag was determined by a two-sample Watson-William test for equal means on the phase distributions in these non-overlapping windows. The gray dashed lines correspond to the best linear fit through the data, and is plotted for illustration purposes when the phase lag is significant.

A) The neurons were divided into a group of cells that were "strongly-tuned" (see Methods) as determined via a one-way ANOVA of the firing response across the stimuli presented in the sequence (p < 0.05). The remaining "weakly-tuned" cells were cells with a weaker, but reliable response as determined by a non-parametric cross-validation analysis. In both groups, the phase lag was significantly negative (left, $F(1,203) = 12.5$, $p<0.001$; right $F(1,699)=52.6$, $p<0.0001$). B) The neurons were split into two groups according to the median phase locking strength. The phase-locking for each cell was determined outside the [preceding, preferred, following] window, since phase precession effects in this window might be expected to negatively affect phase-locking. Thus, the strength of phase-locking was determined based on spikes fired to the preceding-1 and following+1 stimuli (the phase values for these stimuli were similar, Supplementary Figure 2). In both the low and high phase-locking groups, the phase lag was significantly negative (left, $F(1,449) = 17.5$, $p<0.0001$; right $F(1,453)=33.5$, $p<0.0001$). C) Cells were split according to the length of the stimulus sequence (left, $F(1,389) = 5.7$, $p<0.05$; right $F(1,485)=52.6$, $p<0.0001$). D) Cells were split based on the location of the electrodes (left, $F(1,723) = 78.4$, $p<0.0001$; right $F(1,179)<0.01$, $p=0.9$). E) Cells were split into two groups according to the median F-value from a one-way ANOVA of the firing response across all stimuli presented in the sequence (left, $F(1,459) = 9.6$, $p<0.005$; right $F(1,443) = 11.1$, $p<0.001$). F) The data for each cell was split according to the first half or second half of trials (early learning vs. later learning; left, $F(1,903) = 68.9$, $p<0.0001$; right $F(1,903) = 120.3$, $p<0.0001$). G) Stimulus selectivity for each cell was determined by taking the ratio of the mean response to the "preferred" stimulus vs. the mean response to the "other" stimuli. Cells with strong selectivity for their preferred stimulus, such as concept cells (Quiroga, 2012) have a low mean response to the other stimuli and therefore higher selectivity. Cells were split into two groups according to the median value of this measure of stimulus selectivity (left, $F(1,461) = 38.6$, $p<0.0001$; right $F(1,441) = 18.2$, $p<0.0001$). The phase lag tended to be weaker for the more selective cells, but the difference was not significant between the two groups. Source data are provided as a source data file.

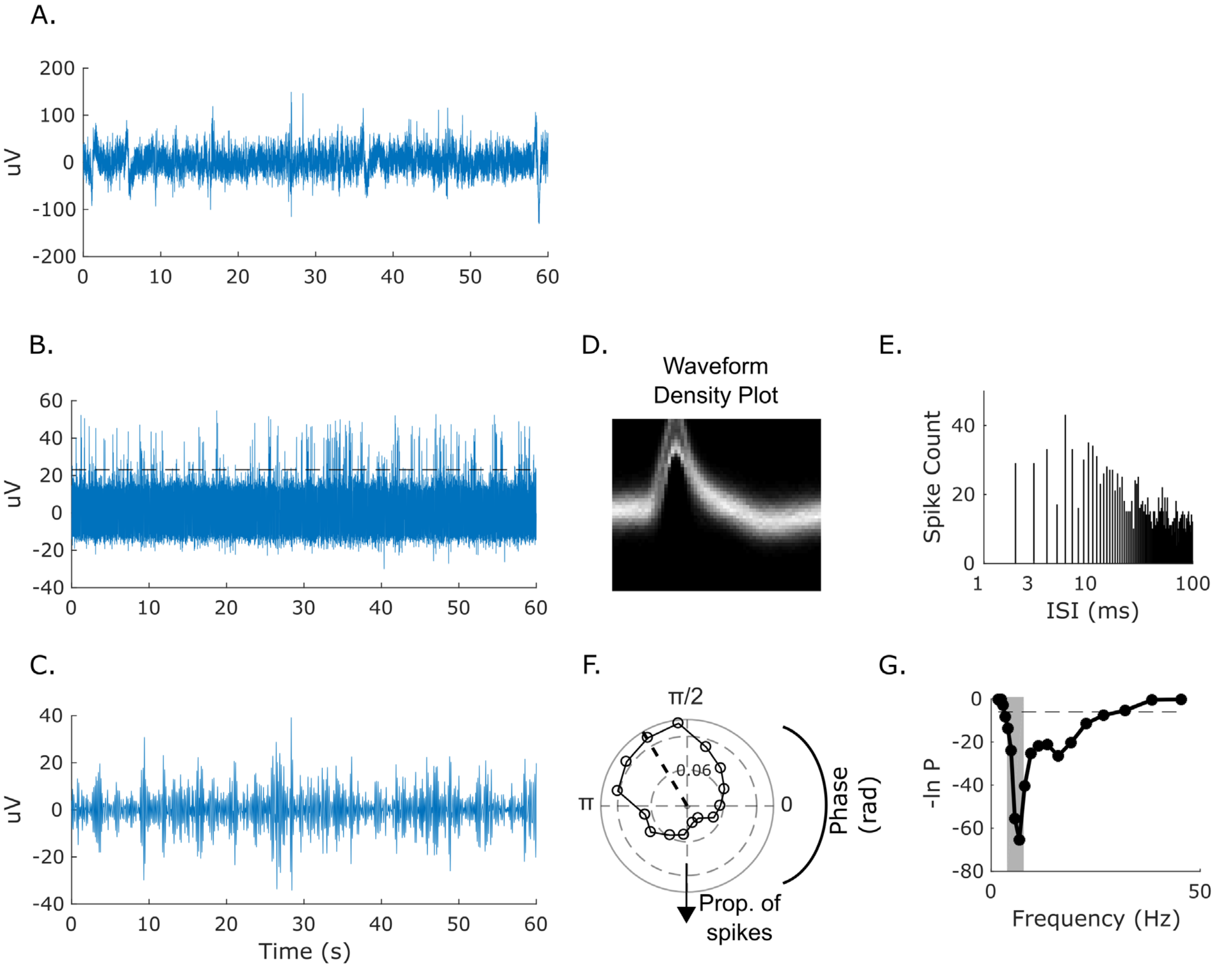

Supplementary Figure 14. Signal recorded from an example microwire. A) Unprocessed signal recorded on one microwire in an epoch of 60s. B) The signal was filtered between 300-3000Hz to extract spikes. The dashed black line corresponds to the threshold used for spike detection. C) The LFP was bandpass filtered between 4-8Hz to extract the phase of each spike. D) Density plot of the spike waveforms. E) Inter-spike interval distribution. F) Phase distribution of spikes in the 4-8Hz theta band. The dashed black line indicates the mean phase for this cell. G) Significance of phase locking (Rayleigh test). The threshold for significance (horizontal black dashed line) is set to $p<0.05$, Bonferroni-corrected for twenty comparisons. The shaded gray area corresponds to the theta band (4-8Hz). It can be seen that this cell showed significant theta phase locking.

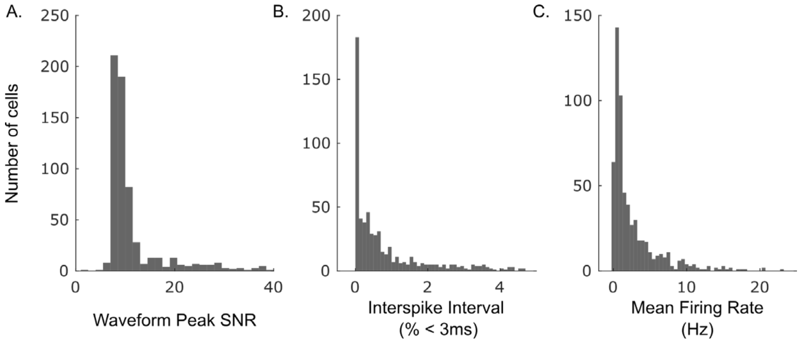

Supplementary Figure 15. Spike quality metrics. A). Histogram of the SNR of the peak of the mean waveform of each unit. B). Histogram of the percentage of interspike intervals that are < 3ms. Note that the large majority of units had less than 1% of interspike intervals < 3 ms. C). Histogram of the mean firing rates.
