# Supplementary material for: Theta-phase dependent neuronal coding during sequence learning in human single neurons
Source: Nat Commun. 2021 Aug 10;12:4839. doi: 10.1038/s41467-021-25150-0 (PMC8355141; doi:10.1038/s41467-021-25150-0)
Supplement: Supplementary file 3 — Reporting summary [file 41467_2021_25150_MOESM3_ESM.pdf]

nature research

Corresponding author(s): Leila Reddy

Last updated by author(s): Jun 23, 2021

Reporting Summary

Nature Research wishes to improve the reproducibility of the work that we publish. This form provides structure for consistency and transparency in reporting. For further information on Nature Research policies, see Authors & Referees and the Editorial Policy Checklist .

Statistics

For all statistical analyses, confirm that the following items are present in the figure legend, table legend, main text, or Methods section.

n/a	Confirmed	
☐	☒	The exact sample size (*n*) for each experimental group/condition, given as a discrete number and unit of measurement
☒	☐	A statement on whether measurements were taken from distinct samples or whether the same sample was measured repeatedly
☐	☒	The statistical test(s) used AND whether they are one- or two-sided<br>*Only common tests should be described solely by name; describe more complex techniques in the Methods section.*
☒	☐	A description of all covariates tested
☐	☒	A description of any assumptions or corrections, such as tests of normality and adjustment for multiple comparisons
☐	☒	A full description of the statistical parameters including central tendency (e.g. means) or other basic estimates (e.g. regression coefficient) AND variation (e.g. standard deviation) or associated estimates of uncertainty (e.g. confidence intervals)
☐	☒	For null hypothesis testing, the test statistic (e.g. *F*, *t*, *r*) with confidence intervals, effect sizes, degrees of freedom and *P* value noted<br>*Give P values as exact values whenever suitable.*
☒	☐	For Bayesian analysis, information on the choice of priors and Markov chain Monte Carlo settings
☒	☐	For hierarchical and complex designs, identification of the appropriate level for tests and full reporting of outcomes
☒	☐	Estimates of effect sizes (e.g. Cohen's *d*, Pearson's *r*), indicating how they were calculated

Our web collection on statistics for biologists contains articles on many of the points above.

Software and code

Policy information about availability of computer code

Data collection	A Neuralynx amplifer was used for signal recording.
Data analysis	The bulk of preprocessing and data analysis was performed with the previously published wave_clus(v. 2019), Fieldtrip(v. 20200315), and Circular Statistics toolbox (v. 2012a) softwares for Matlab. Custom Matlab code was used to generate figures.

For manuscripts utilizing custom algorithms or software that are central to the research but not yet described in published literature, software must be made available to editors/reviewers. We strongly encourage code deposition in a community repository (e.g. GitHub). See the Nature Research guidelines for submitting code & software for further information.

Data

Policy information about availability of data

All manuscripts must include a data availability statement. This statement should provide the following information, where applicable:

- Accession codes, unique identifiers, or web links for publicly available datasets
- A list of figures that have associated raw data
- A description of any restrictions on data availability

Source data for Figures 2, 3, 4, and Supplementary Figures 5, 6, 7, 8 are provided with this paper. Additional data are available from the corresponding author upon reasonable request.

Field-specific reporting

Please select the one below that is the best fit for your research. If you are not sure, read the appropriate sections before making your selection.

Life sciences study design

All studies must disclose on these points even when the disclosure is negative.

Sample size	We recorded neuronal activity from 9 patients. The sample size was 452 units. This sample size is in the range of previously reported studies (e.g., Staresina, Bernhard P., et al. "Recollection in the human hippocampal-entorhinal cell circuitry." Nature communications 10.1 (2019): 1-11. recorded from 238 neurons). This number represents a sufficient sample size in human single neuron studies.
Data exclusions	The study included 9 patients in whom hippocampal and temporal cortical neurons were recorded.
Replication	Single neuron data in humans is a rare dataset. As such we could not provide a replication of the results in a new sample.
Randomization	There were no groups of participants to randomize.
Blinding	Group allocation was not applied in the current study, hence blinding was not necessary.

Reporting for specific materials, systems and methods

We require information from authors about some types of materials, experimental systems and methods used in many studies. Here, indicate whether each material, system or method listed is relevant to your study. If you are not sure if a list item applies to your research, read the appropriate section before selecting a response.

Materials & experimental systems

n/a	Involved in the study
☒	☐ Antibodies
☒	☐ Eukaryotic cell lines
☒	☐ Palaeontology
☒	☐ Animals and other organisms
☐	☒ Human research participants
☒	☐ Clinical data

Methods

n/a	Involved in the study
☒	☐ ChIP-seq
☒	☐ Flow cytometry
☒	☐ MRI-based neuroimaging

Human research participants

Policy information about studies involving human research participants

Population characteristics	Participants were nine patients (four females, age range 18-36 years) suffering from drug-resistant epilepsy.
Recruitment	Participants were recruited solely based on clinical criteria, by a team of clinicians.
Ethics oversight	The Medical Ethics Committee at the VU Medical Center approved the studies.

Note that full information on the approval of the study protocol must also be provided in the manuscript.
